# Individual differences in feelings of certainty surrounding mixed emotions

**DOI:** 10.1371/journal.pone.0332417

**Published:** 2025-11-14

**Authors:** Anthony G. Vaccaro, Shruti Shakthivel, Helen Wu, Rishab Iyer, Jonas Kaplan

**Affiliations:** Brain and Creativity Institute, University of Southern California, Los Angeles, United States of America; Universidad Nacional de Tres de Febrero, ARGENTINA

## Abstract

Ambivalence and uncertainty, though related, are distinct constructs. While research has explored the link between certainty judgements and ambivalence in the context of attitudes and beliefs, little is known about individual differences in how certain people feel about identifying their own ambivalent, or mixed, emotions in the moment. In two samples, we investigated the relationship between the intensity of mixed emotions and self-reported certainty of one’s own current affective experience on a trial-to-trial basis. We additionally tested whether this relationship was moderated by personality and emotional traits. First, in a sample of 140 participants, we found a significant negative relationship between the intensity of mixed feelings and affective certainty, and this relationship was weaker in those with higher emotional intelligence. We next conducted a pre-registered online study with 311 participants in a sample more demographically representative of the United States. We replicated our finding that uncertainty was predicted by higher intensity of co-occurring positive and negative affect, but did not replicate the moderating effect of emotional intelligence. Trait meta-mood, however, did moderate this relationship. Our results show that despite the abstract nature of asking people to report how certain they are of how they feel, and potentially differing interpretations of the question, meaningful variation is found in responses. Future work can refine methods of gauging affective uncertainty, and the implication of affective certainty for mixed emotions on well-being.

## Introduction

If you have ever had the sense that you “are not sure” how you feel, you have experienced *affective uncertainty*. We use the term affective uncertainty to refer to an individual’s *state-level metacognitive assessment* of the identifiability of their own current affective experience. It captures the degree to which someone feels confident that they can recognize and describe what they are feeling in a given moment. This introspective murkiness becomes especially relevant in emotionally complex situations – those that evoke bittersweetness, ambivalence, or internal conflict. Affective certainty represents an intersection between metacognition and emotion. Just as people may be more or less confident in a perceptual or memory judgment, they may also be more or less confident that they can label, articulate, and interpret what they are currently feeling. Though limited, there is some preliminary work on how individuals assign confidence or metacognitive judgements to the identification of their own affective states [1–4]. A recent study showed test-retest reliability within individuals making metacognition judgements on their degree of felt positivity, suggesting promise in using this concept to understand differences between individuals [2].

### Ambivalence and judgements of certainty

A well-established body of research has explored the relationship between ambivalence and certainty, particularly in the domain of attitudes. Previous work has shown that even when subjects report that they hold both positive and negative evaluations of an object (referred to as “objective ambivalence”), this does not necessarily result in a subjective sense of conflict or confusion (“subjective ambivalence”) [5]. The relationship between objective and subjective ambivalence has been shown to vary considerably across individuals and contexts [6–8]. Recent work has extended these findings by showing that the relationship between conflicting emotions or beliefs and subjective ambivalence is moderated by individual differences in tendencies to rely on affective versus cognitive information when forming attitudes [9]. Subjective ambivalence is often measured by directly asking participants how conflicted or ambivalent they feel – probes that often conflate ambivalence with internal uncertainty. The distinction between these two measures has also been used to show that individuals can be quite certain about their mixed or undecided positions [10,11]. Studies have shown that individuals can experience strong mixed or ambivalent reactions while remaining confident that such a conflicted stance is appropriate, especially regarding morally complex scenarios [6,12]. Behavioral studies have shown that certainty can be experimentally manipulated while ambivalence remains stable [13–15], and neuroimaging work has demonstrated that ratings of ambivalence and certainty activate distinct cortical regions [16].

However, most studies have primarily conceptualized certainty in terms of evaluative or propositional attitudes, directed at beliefs or external judgements. These certainty ratings are directed at correctness or validity of the position, rather than at the certainty of self-identification, which have been shown to be distinct aspects of certainty [14,17]. The internal focus distinguishes it from the certainty associated with attitudes, which often involves an evaluation of an external entity (Cohen & Reed, 2006; [18]. This distinction is crucial for understanding the link between feeling uncertain and feeling ambivalent, as prior research has shown that self-related metacognitive judgments are affected differently by context, and related to different cognitive processes, than judgements of beliefs or external objects [19–21]. As such, different traits and sources of information, such as affective knowledge and interoceptive physiology, may influence certainty of self-ambivalent judgements in distinct ways compared to external judgements [21–23]. Yet, the methods and frameworks used to understand ambivalence-certainty links in the domain of attitudes may be fruitfully applied to studying self-related judgements [24], and may be particularly important for understanding links between ambivalence and well-being.

### Trait-level moderators of affective uncertainty

Certainty about one’s affective state can serve as a crucial piece of information in navigating especially emotionally complex states, and how affectively certain one is during complex scenarios may relate to their emotional intelligence [17,25]. The term emotional intelligence has been used to refer to the socio-affective skills, not explained by traditional cognitive intelligence, that help individuals thrive and navigate the problems we face in our daily lives. Research on emotional intelligence has traditionally described the construct as a set of interrelated skills such as perspective-taking, empathy, emotion perception, emotion regulation, and affect labeling [26–30]. These constructs are typically measured with trait-level scales, such as the [31]. These skills may explain differences in how and when individuals generate different affective states, and in what scenarios they judge that their own affective state is appropriate to the context. In addition to general measures of emotional intelligence, several related personal traits may also affect the experience of mental clarity for emotions. For example, alexithymia is a condition characterized by a difficulty in identifying and expressing one’s own feelings, which may be due to insensitivity to the specific meanings behind internal signals from the body or to underdeveloped emotion schemas [32]. Alexithymic traits may be especially related to mixed emotions, which may involve more complex physiological signals, and are known to involve schemas which do not develop till later in development than univalent states [33,34]. Another related concept that may shape the clarity of emotional states is meta-mood. The Trait Meta-Mood Scale (TMMS), developed by Salovey et al. [29], provides a widely used framework for assessing individual differences in meta-emotional processes, or the ways individuals reflect upon and manage their own emotions. The TMMS comprises three core dimensions: Attention to Feelings, Clarity of Feelings, and Mood Repair. Attention to Feelings captures the extent to which individuals notice and value their emotional experiences; Clarity of Feelings reflects how clearly they understand and differentiate their emotional states; and Mood Repair assesses the perceived ability to regulate negative emotions and maintain emotional balance. Together, these constructs offer a nuanced understanding of emotional competence, with implications for mental health, coping strategies, and interpersonal functioning [35].

Overall, confidence in identifying one’s own emotional state is likely a multifaceted process involving physiological interoception, knowledge of emotional labels, and metacognitive processes [36]. The ability to accurately assess one’s own affective uncertainty is a key component of emotional intelligence, allowing individuals to better understand and manage their emotional experiences [29,30]. While these measures are mostly considered trait-level phenomena, there is less work linking them to trial-by-trial state-level self-judgements [35].

### Mixed emotions and affective uncertainty

Research suggests that there may be differences from person to person in how mixed feelings relate to well-being. In some individuals, mixed feelings are related to coping, future improvements in mental health, or progression towards goals; in others, they lead to distress and can even be a sign of worse mental health [37,38] & Wytykowska, 2014; [39] & Traue, 2013; [40–42]. These differences in the effects of mixed feelings may be due to these personally different factors [41,43,44]. It is well-established that there are substantial individual differences in who experiences mixed emotions and how they are experienced [45–47]. Many of these differences arise due to variability in intrinsic personal traits [44,45]. For example, people with higher levels of openness and neuroticism on the ‘Big 5’ personality traits have been found to experience mixed emotions more often in daily life [45,48,49] As mixed feelings can involve different patterns of emotion appraisals, cognitive and personality factors shape the frequency and subjective nature of these experiences across individuals [41,50]. It is also possible that these factors shape whether individuals experience mixed feelings, and believe that they can experience them, through processes which may shape conscious appraisals of valence into bipolar or bivariate representations [33,51,52].

Mixed emotions may be particularly likely to elicit affective uncertainty, as they pose a unique challenge for interpreting one’s own internal state [53,54]. In previous work, we suggested that individuals with less interoceptive accuracy and emotional intelligence may be less likely to experience mixed feelings, and less able to identify how they feel when they do experience them [33]. However, no studies have directly tested the relationship between affective certainty and these traits, especially in the context of complex feelings. Previous work has shown stable trait-level individual differences in attitude certainty (with some level of domain specificity), and as such, there is reason to believe trait-level patterns also explain how individuals judge their own emotional states [24,25]. Given the potential relationship between mental health and how people relate to mixed emotions, there is important theoretical motivation for disentangling these two phenomena. Furthermore, studies on traits such as alexithymia and meta-mood, which are important for the awareness of feelings, have called upon mixed feelings to be an important future direction of study but these studies have not yet been conducted [1,33,41,44,46,48,52,55,56]. With this study, we aimed to understand why the relationship between state-level judgments of affective certainty and the experience of mixed emotions differs across individuals. We hypothesized that traits such as emotional intelligence, alexithymia, and meta-mood clarity would moderate the relationship between emotional complexity and certainty, helping to explain who feels certain versus uncertain when reporting intense objective ambivalence.

## STUDY 1: Piloting of certainty measure and mixed feeling induction/measurement

We first conducted this study with 140 undergraduate student volunteers at University of Southern California, in part to pilot whether subjects would report meaningful variation in affective certainty on a trial by trial basis. Furthermore, we used this initial study to test various possible methods of calculating the “mixed-ness” of feelings that have been used in previous literature [57].

### Hypotheses

H1: We expected a general trend that most people would express less affective certainty when reporting greater mixed feelings. Our subsequent hypotheses focus on how we expect this effect to be moderated by various traits:

H2: Higher self-reported emotional intelligence would lead to a weakening in the negative relationship between ambivalence and certainty, as individuals with higher emotional intelligence may have more experience with complex emotional states

H3: Higher alexithymia scores would lead to a strengthening of the negative relationship between ambivalence and certainty, since mixed feelings as a complex state, and more developmentally advanced schema, may be even more difficult to identify than more typical univalent states.

H4: Higher interoceptive awareness would lead to a weakening of the negative relationship between ambivalence and certainty, since interoceptive ability may help subjects be more confident about their emotional state from being more in touch with ‘bodily evidence’.

## Methods

### Participants

Data was collected from 140 undergraduates (92 female, 48 male; mean age = 20.21, standard deviation = 3.22) at the University of Southern California who received course credit for their participation. 43.6% of subjects identified as Asian, 6.5% as Black, 14.2% as Hispanic or Latino, and 37.1% as White.

### Stimuli selection

The first step of creating a stimulus set was conducting a bulk review of videos to consider including in the pilot study. Our search focused on film noted to induce bittersweet feelings, due to previous work successfully inducing mixed feelings in laboratory settings using bittersweet endings, as well as the general prevalence of the bittersweet trope within film [52,58–60]. The initial search was organized into two categories: live-action film clips and animated videos. Notes were taken on the emotions associated with each clip. Then, we developed a rating system to indicate whether the video elicited bittersweet emotions. Over 300 live-action and 300 animated films were viewed and analyzed, for a total of 603 film clips. Using the rating system, the 603 videos were narrowed down to 85 that achieved a high rating on their ability to elicit bittersweet emotions by a single individual. The 85 selected films were reviewed by a team of 5 that rated each on a scale of 1–5 based on their suitability to function as a good stimulus that elicits ambivalence within viewers. The average of the 5 ratings for each movie was calculated and those with an average rating above 2.5 were chosen as part of the pilot study, narrowing the total number of videos to 47.

### Stimuli

Each subject watched 4–5 videos meant to induce mixed feelings out of 45 potential videos. These video stimuli ranged in length from approximately 2–10 minutes long, and consisted of excerpts from movies, television, and animated short films.

### Procedure

Subjects first completed demographic measures of age, gender identity, race, and ethnicity. Next, each subject was shown 4–5 different videos. After watching each individual video, subjects were asked to complete the following ratings:

1) How familiar were you with this clip before watching? (“Never seen it before”, “Seen it once before”, “Seen it a few times”, “Seen it many times before”)
2) “How certain are you that you know how this video made you feel?” (0%, 20%, 40%, 60%, 8*0%,* 100%).
3) How positive did the clip make you feel (1–5 Likert scale)
4) How negative did the clip make you feel (1–5 Likert scale)
5) How calm-excited did the clip make you feel (1–5 Likert scale)
6) Check off every emotion you felt from the clip? [Happy, Excited, Love, Amusement, Fearful, Sad, Angry, Disgusted, Nostalgic, Bittersweet, Conflicted, Awe]

We chose to assess affective uncertainty through a single global item, rather than through a multiple item scale, to assure subjects would have time to do multiple trials of the task and to prioritize the brevity of the measure in assessing the subject’s current state [44]. Additionally, we specifically made the decision that subjects would rate their certainty prospectively, before giving their exact valence ratings, rather than retrospectively. This was done with the hope of influencing subjects to respond from a general gut feeling of whether they “knew how they felt”, rather than from reflecting retrospectively on whether their specific numerical ratings were “accurate”. We aimed for similarity to the “feeling of knowing” construct from the metacognition literature.

After watching all videos, subjects completed the following measures of individual differences:

1) The Schutte Self Report Emotional Intelligence Scale (SSEIT) [31]- a self-report measure of 4 aspects of emotional intelligence: emotion perception, utilizing emotion, managing self-relevant emotions, and managing other’s emotions
2) The Toronto Alexithymia Scale [32]- a measure of difficulty naming and describing emotions
3) The Multidimensional Assessment of Interoceptive Awareness (MAIA) [61]- a trait measure of introspective ability of one’s own bodily sensations

## Analysis

All analyses were conducted using R version 4.1.1 and the lme4 package [62]. All plots were made with sjPlot [63]. Data from this study is available upon request.

### Measures of mixed feelings

Part of the difficulty in studying mixed feelings is operationalizing their measurement. We used two different measurements of mixed feelings in order to determine their comparability and suitability for gauging the occurrence of mixed feelings on a trial-by-trial basis:

1) An interaction effect between positive and negative valence [64].
2) The Griffin formula for ambivalence: **(Positive+Negative)/2 - |Positive-Negative|** [65]. This theory posits that ambivalence requires two specific conditions to be met. First, ambivalence arises when attitude components have at least moderate intensity, calculated as “(Positive + Negative)/2.” Second, for ambivalence to occur, the magnitudes of the two attitude components must be similar, and this similarity is calculated as “|Pos - Neg|.” As the magnitude difference between the two components increases (meaning the similarity in magnitude decreases), the attitude tends to become more strongly aligned with the more intense component. However, if the similarity is kept constant, ambivalence increases directly with intensity. Therefore, ambivalence is determined by both the similarity in magnitude of the components plus their overall intensity.

### Constructed models

To test each hypothesis, we constructed 2 separate mixed-effects linear models: one for each of the 2 methods of calculating mixed feelings. The outcome variable of every model was the affective certainty during the trial, with the 6 options between 0 and 100% being coded as 1–6. Every model included a random intercept to account for the effect of individual subjects. Models always included positive and negative valence as additional variables to the measure of mixed feelings.

## Results

All of the models had a total of 674 observations at the first level, and 140 groups in the second level of the model: one for each individual subject.

### Uncertainty and mixed feelings

**Fig 1:**
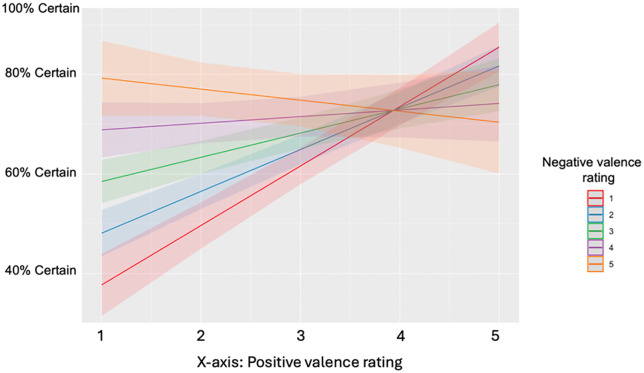
Certainty of affect predicted by positive and negative valence. Predicted certainty of knowing how you feel in a trial, as predicted by positive and negative valence ratings. When positive ratings are high and negative are low, and vice versa, certainty is generally higher. When positive and negative ratings are both high concurrently, certainty begins to decrease.

In both models testing the relationship between mixed feelings and certainty, greater mixed feelings predicted less certainty, whereas increases in both positive and negative feelings independently predicted increased certainty. (Tables 1 and 2; Fig 1)

**Table 1:** Certainty of affect predicted by positive and negative valence, and their interaction.

*Predictors*	*Estimates*	*CI*	*p*
(Intercept)	1.59	1.04–2.15	**<0.001**
Positive	0.77	0.62–0.93	**<0.001**
Negative	0.70	0.51–0.88	**<0.001**
Positive * Negative	−0.18	−0.24 – −0.12	**<0.001**
**Random Effects**			
σ^2^	0.96		
τ_00 Subject_	0.56		
ICC	0.37		
N _Subject_	140		
Observations	674		
Marginal R^2^/ Conditional R^2^	0.143/ 0.458		

Results of mixed effects-model (674 observations, 140 groups) predicting certainty of knowing how you feel. Significance of predictors determined with t-tests using Satterhwaitte’s method.

CI= 95% confidence interval.

**Table 2:** Certainty of affect predicted by positive, negative, and mixed feelings.

*Predictors*	*Estimates*	*CI*	*Statistic*	*p*
(Intercept)	2.33	1.92–2.73	11.20	**<0.001**
Positive	0.50	0.41–0.58	11.43	**<0.001**
Negative	0.45	0.34–0.56	8.11	**<0.001**
Mixed (Griffin)	−0.14	−0.18 – −0.10	−6.82	**<0.001**
**Random Effects**				
σ^2^	0.96			
τ_00_	0.51 _Subject_			
ICC	0.35			
N	140 _Subject_			
Observations	674			
Marginal R^2^/ Conditional R^2^	0.151/ 0.448			

Results of mixed-effects-model (674 observations, 140 subjects) predicting certainty of knowing of how you feel. Significance of predictors determined with t-tests using Satterhwaitte’s method.

CI= 95% confidence interval.

### Individual differences and the relationship between mixed feelings and uncertainty

**Fig 2:**
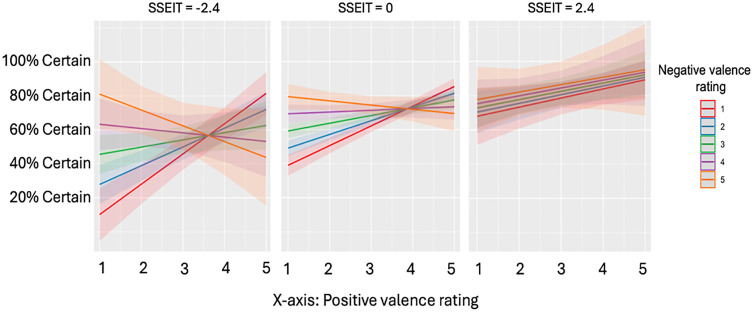
Certainty of affect predicted by valence and low, medium, and high trait emotional intelligence Predicted certainty of knowing how you feel in a trial, as predicted by the subject’s standardized score on the Schutte Self-Report Emotional Intelligence Test (SSEIT) and their positive and negative valence ratings. When positive ratings are high and negative are low, and vice versa, certainty is generally higher. When positive and negative ratings are both high concurrently, certainty begins to decrease, but this effect is lessened in individuals with relatively higher emotional intelligence scores.

While interoception and alexithymia were predictive of certainty in general, they did not have a significant interaction with mixed feelings in any model (S1 and S2 Tables). Emotional intelligence did not significantly interact with the relationship between mixed feelings and certainty in the models using the minimum, gradual threshold, or Griffin methods of calculating mixed feelings (S3 Table). Yet, the three-way interaction between positive feelings, negative feelings, and emotional intelligence significantly predicted increased certainty (Table 3). A closer examination of this result showed that while increasingly intense co-occurring positive and negative feelings were associated with decreases in certainty, higher emotional intelligence led to a weakening of this effect (Fig 2).

**Table 3:** Certainty of affect predicted by valence and emotional intelligence.

*Predictors*	*Estimates*	*CI*	*Statistic*	*p*
(Intercept)	1.69	1.14–2.24	6.03	**<0.001**
Positive	0.75	0.60–0.91	9.71	**<0.001**
Negative	0.68	0.49–0.87	7.19	**<0.001**
SSEIT	0.96	0.41–1.51	3.45	**0.001**
Positive * Negative	−0.18	−0.24 – −0.12	−5.82	**<0.001**
Positive * SSEIT	−0.20	−0.35 – −0.05	−2.64	**0.008**
Negative * SSEIT	−0.23	−0.41 – −0.04	−2.38	**0.017**
(Positive * Negative) * SSEIT	0.07	0.01–0.13	2.23	**0.026**
**Random Effects**				
σ^2^	0.96			
τ_00 Subject_	0.49			
ICC	0.34			
N _Subject_	140			
Observations	674			
Marginal R^2^/ Conditional R^2^	0.201/ 0.472			

Results of mixed-effects-model (674 observations, 140 groups) predicting certainty of knowing of how you feel. Significance of predictors determined with t-tests using Satterhwaitte’s method.

CI= 95% confidence interval. SSEIT= Shutte Self-Report Emotional Intelligence Test.

### Exploring the link between emotional intelligence and certainty of mixed feelings

We next explored the 4 sub-scales of the SSEIT to determine if any specific aspect of emotional intelligence was driving this effect. We found that the Managing Own Emotions subscale and the Utilizing Emotions subscale had the same three-way interaction effect with positive and negative feelings that the total SSEIT score had, whereas the Perceiving Emotions and Managing Other’s Emotions subscales did not (S4 Table).

## Study 2: Pre-registered follow-up study

We decided to investigate whether our findings would replicate in a larger sample more representative of the United States population. A new sample was recruited using the Prolific online subject pool service. In this study, we added a measure of trait meta-mood, to investigate whether trait-levels of emotional awareness were related to the trial-by-trial measure of uncertainty we measured. We also reduced the sample of videos to those most effective at inducing positive, negative, or mixed feelings, based on ratings from the first study’s pool of participants, by removing videos that consistently received ratings of 1 and 2 in both valence categories

We pre-registered this follow-up study at OSF Registries (https://osf.io/anbvj). After pre-registration, we additionally tested the effect of adding a random effect for stimulus to the models, but this did not change the significance of any predictors in the analyses.

### Pre-registered hypotheses

Hypothesis 1: Participants will be more uncertain of how they feel when reporting a co-occurrence of positive and negative valence (mixed feelings).

Hypothesis 2a: Participants with higher emotional intelligence will exhibit a weaker relationship between mixed feelings and uncertainty.

Hypothesis 2b: Higher scores on the “managing self-relevant emotions” sub-scale of the emotional intelligence scale specifically will lead to a weaker relationship between mixed feelings and uncertainty.

Hypothesis 3: The three measures of the meta-mood scale (Clarity, Repair, and Attention) will all lead to a weaker relationship between mixed feelings and uncertainty.

## Methods

### Ethics

This study was approved by the Institutional Review Board at the University of Southern California. Written informed consent was from subjects before participation.

### Participants

Data was collected on Prolific from 311 subjects (149 female, 154 male, 5 non-binary, 2 who reported ‘other’, and 1 who preferred not to say) in the United States with ages ranging from 18–65 (mean = 35.08, standard deviation = 11.82). 1.6% of subjects identified as American Indian, 10% as Asian, 15.1% as Black, 12.5% as Hispanic or Latino, 0.6% as Native Hawaiian or Other Pacific Islander, and 68.8% as White. Subjects were paid $7.50 for the 50 minute study. All data was obtained on October 7th, 2022.

### Procedure

Each subject watched 4 random videos meant to induce mixed feelings out of 28 potential videos, with each video being evenly distributed across the total sample. Valence and emotional category ratings for these stimuli are included at the end of the Supplementary Materials. After each video, subjects answered the same questions as the previous study.

After watching all videos, subjects completed the following measures of individual differences, the first two for our pre-registered hypotheses, and the following 2 for exploratory purposes:

1) The Schutte Self-Report Emotional Intelligence Scale
2) The Trait Meta-Mood Scale [29]- A measure of various traits related to identifying, understanding, and reflecting on one’s own mood
3) The Multidimensional Assessment of Interoceptive Awareness
4) The Ten-Item Personality Index [66]

## Analysis

This dataset is publicly available at https://osf.io/2t43c.

### Data exclusion

Following pre-registered procedures, subjects’ data was excluded entirely if their total time taking the survey was less than the total length of all the videos they watched. Observations were excluded from the Hypothesis 1 analyses if any of certainty, positive valence, or negative valence is missing. Additionally, in all other analyses, subjects’ data were excluded from the analysis if they did not complete the entire relevant scale. 43 subjects clicked to start the study but did not complete it, and 7 subjects timed out and did not complete the study. Data was automatically collected till 311 participants completed the survey.

### Measures of mixed feelings

We used two measures of mixed feelings: the interaction between positive and negative valence, and the Griffin formula.

## Results

### Uncertainty and mixed feelings

**Fig 3:**
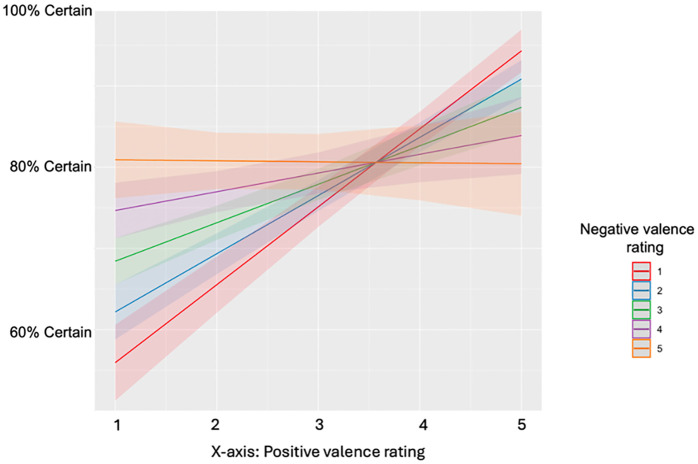
Certainty of affect predicted by positive and negative valence (study 2) Predicted certainty of knowing how you feel in a trial, as predicted by positive and negative valence ratings. When positive ratings are high and negative are low, and vice versa, certainty is generally higher. When positive and negative ratings are both high concurrently, certainty begins to decrease.

Subjects reported a range of positive-negative valence combinations across trials, ranging from purely univalent to intense ambivalence (see S1 Fig). We again found that mixed feelings had a significant negative relationship with certainty in both models, whereas positive and negative feelings individually were positively associated with certainty (Fig 3; Table 4).

**Table 4:** Certainty of affect predicted by positive, negative, and mixed feelings (Study 2).

	Model 1 (Pos-Neg Interaction)				Model 2 (Griffin_Mix)			
*Predictors*	*Estimates*	*CI*	*Statistic*	*p*	*Estimates*	*CI*	*Statistic*	*p*
(Intercept)	2.88	2.48–3.29	14.02	**<0.001**	3.59	3.30–3.88	24.08	**<0.001**
Positive	0.60	0.50–0.70	11.88	**<0.001**	0.35	0.29–0.40	12.56	**<0.001**
Negative	0.43	0.31–0.56	6.86	**<0.001**	0.20	0.14–0.27	6.04	**<0.001**
Positive * Negative	−0.12	−0.16 – −0.09	−6.59	**<0.001**				
Mixed (Griffin)					−0.19	−0.24 – −0.14	−8.07	**<0.001**
**Random Effects**								
σ^2^	0.73				0.72			
τ_00_	0.40 _Subject_				0.39 _Subject_			
ICC	0.36				0.35			
N	311 _Subject_				311 _Subject_			
Observations	1237				1237			
Marginal R^2^/ Conditional R^2^	0.127/ 0.438				0.140/ 0.443			

Results of mixed-effects-model (1237 observations, 311 subjects) predicting certainty of knowing of how you feel. CI = 95% confidence intervals. Significance of variables determined with t-tests.

### Individual differences and the relationship between mixed feelings and uncertainty

**Fig 4:**
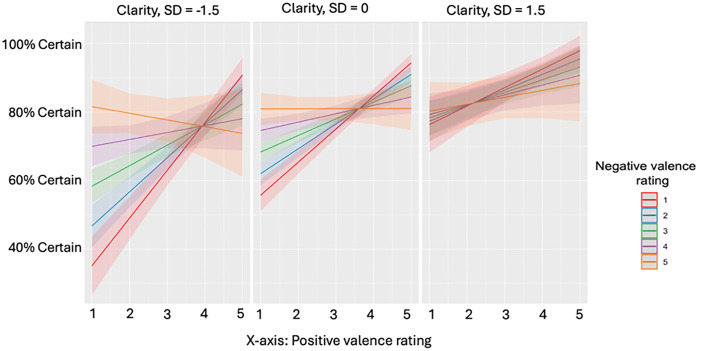
Certainty of affect predicted by valence and low, medium, and, high trait emotional clarity Predicted certainty of knowing how you feel in a trial, as predicted by the subject’s standardized Clarity score on the Trait Meta-Mood Scale (at −1.5 standard deviations from the mean, the mean, and 1.5 standard deviation above the mean) and their positive and negative valence ratings. When positive ratings are high and negative are low, and vice versa, certainty is generally higher. When positive and negative ratings are both high concurrently, certainty begins to decrease, but this effect is lessened in individuals with relatively higher clarity scores.

While emotional intelligence itself was predictive of certainty, there was no significant interaction with mixed feelings in this sample (Table 5). The managing one’s own emotions subscale was also not a significant predictor, nor was its interaction with mixed feelings (S5 Table). Of the meta-mood subscales, the clarity subscale had a significant interaction with mixed feelings, in which high scores were associated with a decrease in the negative relationship between mixed feelings and certainty (Fig 4; Table 6). Attention and mood repair did not have a significant interaction effect (S6 Tables).

**Table 5:** Certainty of affect predicted by valence and emotional intelligence (SSEIT).

	Model 1 (Pos-Neg Interaction)				Model 2 (Griffin_Mix)			
*Predictors*	*Estimates*	*CI*	*Statistic*	*p*	*Estimates*	*CI*	*Statistic*	*p*
(Intercept)	2.99	2.58–3.40	14.28	**<0.001**	3.66	3.37–3.95	24.46	**<0.001**
Positive	0.58	0.48–0.68	11.13	**<0.001**	0.33	0.28–0.39	11.90	**<0.001**
Negative	0.42	0.29–0.54	6.44	**<0.001**	0.20	0.13–0.27	5.79	**<0.001**
SSEIT	0.47	0.09–0.86	2.43	**0.015**	0.36	0.08–0.65	2.48	**0.013**
Positive * Negative	−0.12	−0.16 – −0.08	−6.28	**<0.001**				
Positive * SSEIT	−0.08	−0.18–0.02	−1.62	0.105				
Negative * SSEIT	−0.08	−0.20–0.03	−1.42	0.157				
(Positive * Negative) * SSEIT	0.02	−0.01–0.06	1.29	0.197				
Mixed (Griffin)					−0.19	−0.23 – −0.14	−7.76	**<0.001**
Mix * SSEIT					0.01	−0.04–0.05	0.39	0.695
SSEIT * Positive					−0.04	−0.10–0.01	−1.49	0.138
SSEIT* Negative					−0.03	−0.09–0.04	−0.85	0.395
**Random Effects**								
σ^2^	0.73				0.72			
τ_00_	0.37 _Subject_				0.37 _Subject_			
ICC	0.34				0.34			
N	311 _Subject_				311 _Subject_			
Observations	1237				1237			
Marginal R^2^/ Conditional R^2^	0.166/ 0.450				0.177/ 0.455			

Results of mixed-effects-model (1237 observations, 311 subjects) predicting certainty of knowing of how you feel. Significance of predictors determined with t-tests using Satterhwaitte’s method.

CI= 95% confidence interval. SSEIT= Shutte Self-Report Emotional Intelligence Test.

**Table 6:** Certainty of affect predicted by valence and trait clarity.

	Model 1 (Pos-Neg Interaction)				Model 2 (Griffin_Mix)			
*Predictors*	*Estimates*	*CI*	*Statistic*	*p*	*Estimates*	*CI*	*Statistic*	*p*
(Intercept)	2.87	2.47–3.27	14.02	**<0.001**	3.57	3.28–3.86	24.21	**<0.001**
Positive	0.60	0.50–0.70	11.88	**<0.001**	0.35	0.30–0.40	12.72	**<0.001**
Negative	0.44	0.31–0.56	6.86	**<0.001**	0.20	0.14–0.27	6.04	**<0.001**
Clarity	1.06	0.66–1.46	5.22	**<0.001**	0.71	0.42–1.01	4.70	**<0.001**
Positive * Negative	−0.12	−0.16 – −0.08	−6.48	**<0.001**				
Positive * Clarity	−0.19	−0.29 – −0.10	−3.85	**<0.001**				
Negative * Clarity	−0.23	−0.35 – −0.11	−3.77	**<0.001**				
(Positive * Negative) * Clarity	0.05	0.02–0.09	2.85	**0.004**				
Mixed (Griffin)					−0.18	−0.23 – −0.13	−7.62	**<0.001**
Mix * Clarity					0.05	0.01–0.10	2.26	**0.024**
Clarity* Positive					−0.08	−0.14 – −0.03	−2.85	**0.004**
Clarity * Negative					−0.11	−0.18 – −0.05	−3.33	**0.001**
**Random Effects**								
σ^2^	0.73				0.72			
τ_00_	0.33 _Subject_				0.33 _Subject_			
ICC	0.31				0.31			
N	308 _Subject_				308 _Subject_			
Observations	1225				1225			
Marginal R^2^/ Conditional R^2^	0.188/ 0.442				0.197/ 0.449			

Results of mixed-effects-model (1225 observations, 308 subjects) predicting certainty of knowing of how you feel. Significance of variables determined with t-tests using Satterhwaitte’s method.

CI= 95% confidence interval.

### Exploratory analyses of the Big 5

Extraversion, Openness, and Emotional Stability did not significantly predict certainty in any model (S7-S9 Tables). Agreeableness and Conscientiousness significantly predicted certainty, but did not significantly interact with positive, negative, or mixed valence (S10 Table). Greater Conscientiousness significantly predicted greater certainty (also S10 Table). Additionally, there was a significant negative interaction between negativity and conscientiousness in predicting certainty (S11 Table).

## Discussion

### Mixed feelings and affective certainty

Consistently in both samples, we find that the intensity of mixed feelings is associated with affective uncertainty. Specifically, as concurrent positive and negative feelings increase in intensity, participants report less certainty in identifying how they feel. While prior work has explored the relationship between attitudinal ambivalence and certainty, often focusing on certainty about beliefs or external evaluations [8,67,68] our study specifically examined a state-level metacognitive assessment of the identifiability of one’s *own* current emotional experience. Our data reveals meaningful variability in this affective certainty, particularly in the context of mixed emotions. Our results underscore that individuals differ in their confidence in identifying their feelings, and importantly, this variability is partially explained by individual trait differences.

### Emotional intelligence

In Study 1, emotional intelligence as measured by the SSEIT interacted with the negative association between certainty and mixed feelings. Specifically higher emotional intelligence was associated with a weaker version of the effect. The effect was mainly driven by the managing emotion subscale of the SSEIT. However, this finding wasn’t replicated in our pre-registered analysis – which differed demographically in terms of gender, age, and race.

A recent study had a seemingly contradictory finding concerning mixed emotions and emotional intelligence: higher emotional intelligence was associated with experiencing less mixed feelings in workplace scenarios, and this effect was mostly driven by the Managing Emotion ability [69] The authors suggest that the relationship is driven by high emotion-management down-regulating conflicting affect, leading to more univalent states. In our study, we specifically aimed to induce mixed feelings. It is possible that individuals with high emotional intelligence do often regulate mixed feelings away, but when scenarios truly call for them, they are more certain that they feel them, and that they *should* be feeling them. It is also possible that our paradigm, using popular media, induced different types of mixed feelings than in the aforementioned study. Mixed feelings induced by different contexts may involve different types of interactions between emotional and cognitive/appraisal processes [41,44,50,70–72]. It is possible that these various instances of mixed feelings have different relationships to emotional intelligence.

### Trait meta-mood and trial-by-trial certainty

In our second study we found that the clarity sub-scale of the Trait Meta-Mood scale interacted with the negative relationship between mixed feelings and certainty, in line with prior work [29]. Our results demonstrate a link between two similar constructs: certainty and clarity. Our results suggest that our trial-by-trial certainty measure capturing the state-level self judgement is probing the same cognitive function that is traditionally assessed on a trait-level basis. The clarity sub-scale has frequently been found to blunt the negative effect of stressors on general and life satisfaction [73–75]. Interestingly, many studies on mixed feelings have focused on life satisfaction, often finding positive effects [37,76]; while others have found vast individual differences and even negative effects [41,42,77–80]. Clarity may be an important factor in explaining these individual differences, where reported mixed feelings that stem from a lack of clarity about one’s affective state are related to distress, whereas clear mixed feelings can be a sign of processing complicated affective scenarios. Previous studies have found that mixed feelings which subjects feel certain about are more stable overtime than ones for which they feel uncertain [12,16,81]. Future studies could test whether this holds true with a paradigm where subjects view the stimuli again on later dates. This could give an additional measure of attitude stability, as well as be used to investigate whether uncertain subjects may become more certain of ambivalent feelings overtime with additional processing of the stimulus. In this way, the clarity trait could represent a speed of processing complex affective scenarios, leading to less occurrence of uncertain feelings in daily life.

### Age and cultural effects on mixed emotions

The similarities and differences between the results of our first study and our pre-registered study demonstrate the importance of understanding which emotion-trait relationships are generalizable across populations, versus which may differ based on social and demographic factors [44,82,83]. The interaction between emotional intelligence, certainty, and mixed feelings did not replicate in the sample that was demographically representative of the United States population. It is possible that the relationships between measures of emotional intelligence, affective certainty, and mixed feelings were strongly related to the specific demographic factors of our first sample. Specifically, our sample of undergraduate students was much younger than the replication sample, and had a plurality of subjects identifying as Asian, whereas the replication sample had a majority of subjects identifying as White. Previous research has found significant differences in the experience of, and comfort with, mixed emotions between groups of different ages [71,84], and cultural backgrounds [80,83,85–87]. These findings have included younger adults having less comfort with mixed feelings, as well as Asian-American and people natively from Asian countries having more comfort with mixed feelings. The interplay between these demographic factors in our first sample may lead to emotional intelligence explaining more of the variability in how strong the relationship between uncertainty and mixed feelings is. Future studies with larger samples should investigate how demographic factors, and interactions between them, affect these complex relationships between traits, feelings, and certainty, especially in an international sample.

### Implications for future research on mixed emotions

Our results suggest several directions for future work on mixed emotions. Affective uncertainty has promise in shedding light on individual variations in mixed feelings, brought about by different traits and cognitive abilities. Given the established literature on traits that moderate certainty in ambivalent attitudinal judgments, it would be valuable to directly test which of these same traits overlap or differ with those that moderate certainty about one’s own affective state [8,12,24,36,44,88,89]. Testing both judgements in the same subjects could further illuminate the similarities and differences between metacognitive processes applied to beliefs versus internal feelings. Additionally, this format could be used for testing whether in the moment manipulations, such as changes to presented information and context surrounding the positive and negative aspects of the stimuli, can influence affective certainty in a similar manner to manipulations to subjective ambivalence [13,16].

To deepen our understanding of these connections, future studies should explore affective certainty during mixed emotional experiences across a wider range of contexts [44,89,90], moving beyond film-induced emotions to capture mixed feelings encountered in daily life and in response to various types of meaningful events [91,92]. This is especially important, as we know that real-world scenarios, such as contexts that force decision-making, can increase the link between mixed emotions and psychological distress [68,93]. On this note, affective uncertainty for mixed emotions may be particularly relevant to various psychiatric disorders, explaining difficulties in handling complex affective situations [1,36].

Some of the variance in our data could be related to how subjects interpret the certainty question differently. Cognitive processes and individual lay theories about emotion may influence how subjects assess and report the identifiability of their feelings, especially in the face of ambivalence [51,52,94]. Future work could explicitly investigate how individuals understand and interpret the concept of affective uncertainty, as this in itself could provide valuable insights into the factors that contribute to varying subjective experiences of emotional clarity and ambivalence. However, despite this potential source of variability in interpretation, our finding of a consistent negative relationship between the intensity of mixed feelings and affective certainty, replicating across two demographically different samples, underscores that our measure is capturing a meaningful and generalizable phenomenon related to the confidence in identifying one’s own mixed emotional state.

## Conclusions

This study demonstrates variation in the experiences of mixed feelings both between subjects, and between individual trials. Our results support the commonly observed link between experiencing ambivalence and a sense of uncertainty within the domain of self-related judgements, specifically showing that higher intensity of co-occurring positive and negative affect is associated with lower affective certainty. Importantly, our analyses reveal that this relationship is not uniform; various individual traits moderate this effect, indicating that some individuals are indeed able to experience intense mixed feelings while still feeling fully certain about the identifiability of their affective state. These findings suggest that affective certainty ratings provide a meaningful insight into self-awareness for affect, particularly in complex emotional scenarios.With this baseline knowledge, more advanced techniques of measuring self-awareness for emotion can be developed. Mixed feelings are a prime candidate for demonstrating the important interplay between metacognitive processes, personalized traits, and emotion: these relationships should be studied further. Finally, these results may demonstrate a broad generalizability of this uncertainty-mixed feelings association, though at different strengths depending on various factors. We may have a natural tendency to *feel* that positivity and negativity do not go together, even when we are, in fact, experiencing them.

## Supporting information

S1 Fig Counts of positive and negative valence ratings across 1237 trials Matrix displays counts of each possible combination of positive and negative valence rating across all subjects in Study 2. (DOCX)

S1 Appendix Supplemental Results Tables from Study 1 Results of mixed-effects-models (674 observations, 140 subjects) predicting certainty of knowing of how you feel from valence and and personality measures. Significance of predictors determined with t-tests using Satterhwaitte’s method CI = 95% confidence interval.
(DOCX)

S2 Appendix Supplemental Results Tables from Study 2 Results of mixed-effects-models (1233–1237 observations, 310–311 subjects) predicting certainty of knowing of how you feel from valence and personality measures. Significance of predictors determined with t-tests using Satterhwaitte’s method CI = 95% confidence interval.
(DOCX)

S3 Appendix Stimuli Ratings Across Both Studies Subject (N = 451 ratings across both studies for video stimuli. Each subject rated 4–5 videos.
(DOCX)
