# Supplementary figures and images for: Individual differences in feelings of certainty surrounding mixed emotions
Source: PLoS One. 2025 Nov 14;20(11):e0332417. doi: 10.1371/journal.pone.0332417 (PMC12617922; doi:10.1371/journal.pone.0332417)

**Figure S1: Counts of positive and negative valence ratings across 1237 trials**


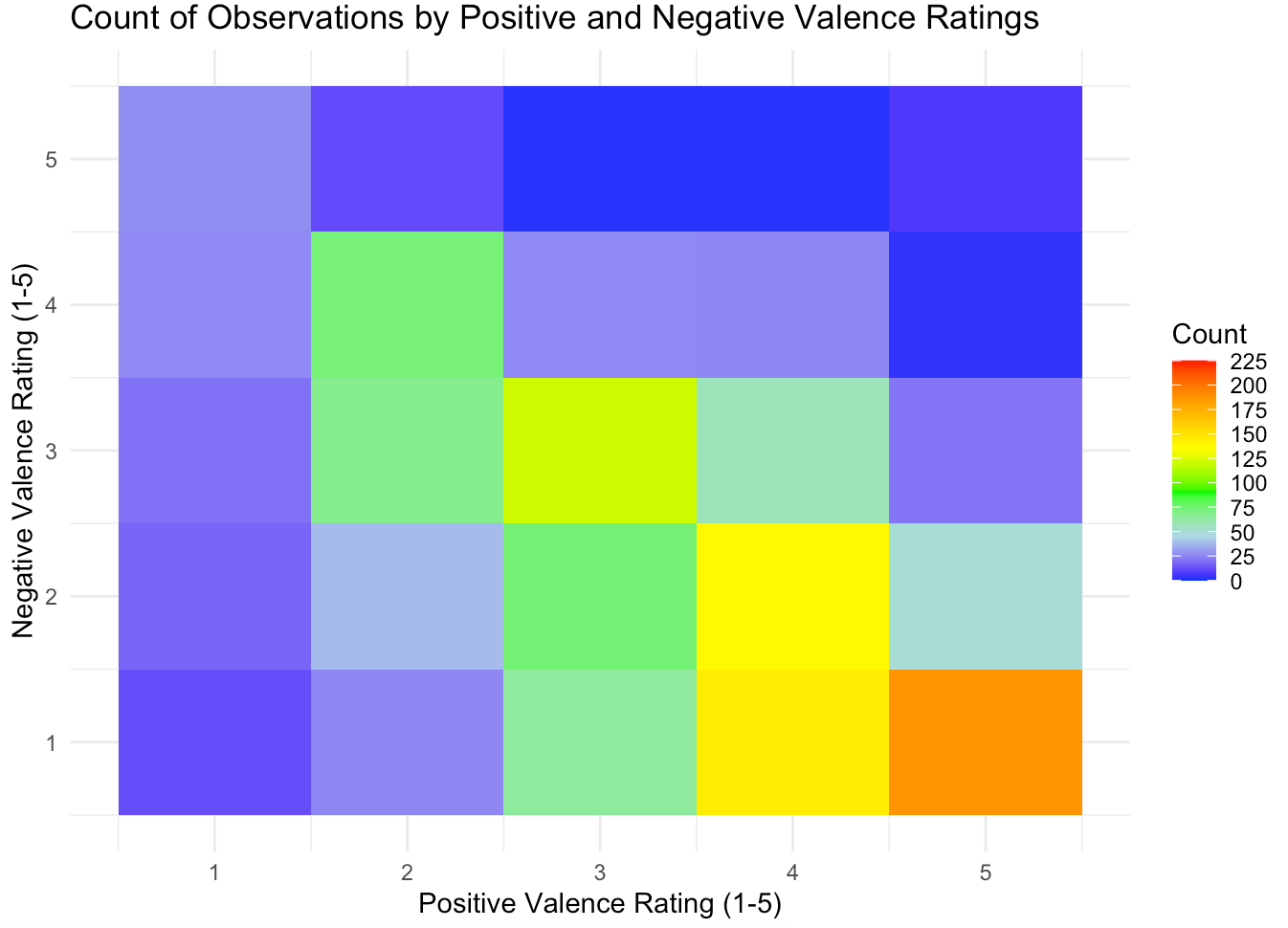

Supplement: S1 Fig — (DOCX) [file pone.0332417.s001.docx]
