# Supplementary material for: Individual differences in feelings of certainty surrounding mixed emotions
Source: PLoS One. 2025 Nov 14;20(11):e0332417. doi: 10.1371/journal.pone.0332417 (PMC12617922; doi:10.1371/journal.pone.0332417)
Supplement: S1 Appendix — Significance of predictors determined with t-tests using Satterhwaitte’s method CI = 95% confidence interval. (DOCX) [file pone.0332417.s002.docx]

**Appendix S1: Supplementary Results Tables from Study 1**

**Certainty of affect predicted by valence and alexithymia (TAS)**

|  | **Model 1 (Pos-Neg Interation)** | | | | **Model 2(Mixed_Griffin)** | | | |  |  |  |  |  |  |
| --- | --- | --- | --- | --- | --- | --- | --- | --- | --- | --- | --- | --- | --- | --- |
| *Predictors* | *Estimates* | *CI* | *Statistic* | *p* | *p* | *Estimates* | *CI* | *Statistic* | *p* |  |  |  |  |  |
| (Intercept) | 1.83 | 1.26 – 2.40 | 6.29 | **<0.001** | **<0.001** | 2.79 | 2.39 – 3.19 | 13.67 | **<0.001** |  |  |  |  |  |
| Positive | 0.72 | 0.56 – 0.88 | 8.97 | **<0.001** | **<0.001** | 0.38 | 0.29 – 0.46 | 9.02 | **<0.001** |  |  |  |  |  |
| Negative | 0.65 | 0.46 – 0.84 | 6.73 | **<0.001** | **<0.001** | 0.33 | 0.23 – 0.42 | 6.85 | **<0.001** |  |  |  |  |  |
| TAS | -0.69 | -1.27 – -0.11 | -2.32 | **0.021** | **0.019** | -0.49 | -0.89 – -0.08 | -2.36 | **0.019** |  |  |  |  |  |
| Positive * Negative | -0.17 | -0.23 – -0.11 | -5.40 | **<0.001** |  |  |  |  |  |  |  |  |  |  |
| Positive * TAS | 0.14 | -0.02 – 0.31 | 1.74 | 0.082 |  |  |  |  |  |  |  |  |  |  |
| Negative * TAS | 0.11 | -0.08 – 0.30 | 1.15 | 0.251 |  |  |  |  |  |  |  |  |  |  |
| (Positive * Negative) * TAS | -0.03 | -0.09 – 0.03 | -1.14 | 0.257 |  |  |  |  |  |  |  |  |  |  |
| TAS * Min |  |  |  |  | 0.545 |  |  |  |  |  |  |  |  |  |
| TAS * Positive |  |  |  |  | 0.082 | 0.08 | -0.01 – 0.16 | 1.75 | 0.081 |  |  |  |  |  |
| TAS * Negative |  |  |  |  | 0.516 | 0.03 | -0.07 – 0.12 | 0.57 | 0.572 |  |  |  |  |  |
| Mixed Griffin |  |  |  |  |  | -0.24 | -0.31 – -0.16 | -6.39 | **<0.001** |  |  |  |  |  |
| TAS * Mixed Griffin |  |  |  |  |  | -0.02 | -0.10 – 0.05 | -0.61 | 0.545 |  |  |  |  |  |
| **Random Effects** | | | | | | | | | | | | | | |
| σ^2^ | 0.96 | | | | 0.95 | | | |  |  |  |  |  |  |
| τ_00_ | 0.53 _Subject_ | | | | 0.50 _Subject_ | | | |  |  |  |  |  |  |
| ICC | 0.36 | | | | 0.35 | | | |  |  |  |  |  |  |
| N | 136 _Subject_ | | | | 136 _Subject_ | | | |  |  |  |  |  |  |
| Observations | 654 | | | | 654 | | | |  |  |  |  |  |  |
| Marginal R^2^ / Conditional R^2^ | 0.174 / 0.468 | | | | 0.182 / 0.464 | | | |  |  |  |  |  |  |

**Certainty of affect predicted by valence and interoceptive awareness (MAIA)**

|  | **Model 1 (Pos-Neg Interaction)** | | | | **Model 2 (Mixed_Griffin)** | | | | | | | | | | |  |  |  |  |  |  |  |  |  |  |
| --- | --- | --- | --- | --- | --- | --- | --- | --- | --- | --- | --- | --- | --- | --- | --- | --- | --- | --- | --- | --- | --- | --- | --- | --- | --- |
| *Predictors* | *Estimates* | *CI* | *Statistic* | *p* | *Estimates* | | | *CI* | | | *Statistic* | | | *p* | | | | | |  |  |  |  |  |  |
| (Intercept) | 1.90 | 1.31 – 2.48 | 6.37 | **<0.001** | 2.85 | | | 2.43 – 3.26 | | | 13.46 | | | **<0.001** | | | |  |  |  |  |  |  |  |  |
| Positive | 0.71 | 0.56 – 0.87 | 8.83 | **<0.001** | 0.36 | | | 0.28 – 0.45 | | | 8.37 | | | **<0.001** | | | |  |  |  |  |  |  |  |  |
| Negative | 0.64 | 0.45 – 0.83 | 6.54 | **<0.001** | 0.32 | | | 0.22 – 0.41 | | | 6.53 | | | **<0.001** | | | |  |  |  |  |  |  |  |  |
| MAIA | 0.43 | -0.10 – 0.96 | 1.58 | 0.114 | 0.10 | | | -0.16 – 0.35 | | | 0.75 | | | 0.452 | | | |  |  |  |  |  |  |  |  |
| Positive * Negative | -0.17 | -0.23 – -0.11 | -5.46 | **<0.001** |  | |  | | |  | | | | |  | | | | | |  |  | |  |  |
| Positive * MAIA | -0.09 | -0.24 – 0.06 | -1.15 | 0.252 |  | |  | | |  | | | | |  | | | | | |  |  | |  |  |
| Negative * MAIA | -0.07 | -0.25 – 0.11 | -0.79 | 0.430 |  | |  | | |  | | | | |  | | | | | |  |  | |  |  |
| (Positive * Negative) * MAIA | 0.03 | -0.03 – 0.09 | 0.83 | 0.408 |  | |  | | |  | | | | |  | | | | | |  |  | |  |  |
| Positive * TAS |  |  |  |  | -0.02 | | | -0.07 – 0.02 | | | -0.99 | | | 0.324 | | | |  |  |  |  |  |  |  |  |
| Negative * TAS |  |  |  |  |  | | |  | | |  | | |  | | | |  |  |  |  |  |  |  |  |
| Mixed Griffin |  |  |  |  | -0.25 | | | -0.32 – -0.17 | | | -6.49 | | | **<0.001** | | | |  |  |  |  |  |  |  |  |
| MAIA * Mixed Griffin |  |  |  |  | 0.00 | | | -0.07 – 0.07 | | | 0.01 | | | 0.988 | | | |  |  |  |  |  |  |  |  |
| MAIA * Negative |  |  |  |  | 0.01 | | | -0.07 – 0.10 | | | 0.33 | | | 0.739 | | | |  |  |  |  |  |  |  |  |
| MAIA * Positive |  |  |  |  |  |  | | |  | | |  | | | | |  | |  | | | |  | |  |
| σ^2^ | 0.94 | | | | 0.94 | | | | | | | |  |  |  |  |  |  |  |  |  |  |  |  |  |
| τ_00_ | 0.56 _Subject_ | | | | 0.52 _Subject_ | | | | | | | |  |  |  |  |  |  |  |  |  |  |  |  |  |
| ICC | 0.37 | | | | 0.36 | | | | | | | |  |  |  |  |  |  |  |  |  |  |  |  |  |
| N | 129 _Subject_ | | | | 128 _Subject_ | | | | | | | |  |  |  |  |  |  |  |  |  |  |  |  |  |
| Observations | 620 | | | | 615 | | | | | | | |  |  |  |  |  |  |  |  |  |  |  |  |  |
| Marginal R^2^ / Conditional R^2^ | 0.156 / 0.473 | | | | 0.170 / 0.466 | | | | | | | |  |  |  |  |  |  |  |  |  |  |  |  |  |

**Table S3: Certainty of affect predicted by mixed feelings and emotional intelligence**

|  | **Griffin_Mix Model** | | | |  |
| --- | --- | --- | --- | --- | --- |
| *Predictors* | *Estimates* | *CI* | *Statistic* | *p* |  |
| (Intercept) | 2.70 | 2.32 – 3.09 | 13.73 | **<0.001** |  |
| SSEIT | 0.58 | 0.18 – 0.97 | 2.85 | **0.005** |  |
| Positive | 0.39 | 0.31 – 0.47 | 9.70 | **<0.001** |  |
| Negative | 0.33 | 0.24 – 0.43 | 7.13 | **<0.001** |  |
| SSEIT * Positive | -0.06 | -0.14 – 0.01 | -1.61 | 0.107 |  |
| SSEIT * Negative | -0.07 | -0.16 – 0.02 | -1.47 | 0.143 |  |
| Griffin_Mix | -0.24 | -0.32 – -0.17 | -6.60 | **<0.001** |  |
| SSEIT * Griffin_Mix | 0.05 | -0.02 – 0.12 | 1.37 | 0.170 |  |
| **Random Effects** | | | | | |
| σ^2^ | 0.96 | | | |  |
| τ_00_ | 0.46 _Subject_ | | | |  |
| ICC | 0.32 | | | |  |
| N | 140 _Subject_ | | | |  |
| Observations | 674 | | | |  |
| Marginal R^2^ / Conditional R^2^ | 0.207 / 0.463 | | | |  |

**Table S4: Certainty of affect predicted by valence and emotional intelligence (SSEIT) subscales**

|  | **Managing Own Emotion Model** | | | | **Managing Others Emotion Model** | | | | **Utilizing Emotion Model** | | | | | | **Emotion Perception Model** | | | | | | | |  |
| --- | --- | --- | --- | --- | --- | --- | --- | --- | --- | --- | --- | --- | --- | --- | --- | --- | --- | --- | --- | --- | --- | --- | --- |
| *Predictors* | *Estimates* | *CI* | *Statistic* | *p* | *Estimates* | *CI* | *Statistic* | *p* | *Estimates* | *CI* | *Statistic* | | *p* | | *Estimates* | | *CI* | | *Statistic* | | *p* | |  |
| (Intercept) | 1.68 | 1.13 – 2.24 | 5.96 | **<0.001** | 1.68 | 1.13 – 2.23 | 5.97 | **<0.001** | | 1.63 | 1.08 – 2.18 | 5.79 | | **<0.001** | | 1.69 | | 1.13 – 2.24 | | 5.96 | | **<0.001** | |
| SSEIT OwnEmotion | 0.88 | 0.34 – 1.42 | 3.21 | **0.001** |  |  |  |  | |  |  |  | |  | |  | |  | |  | |  | |
| Positive | 0.75 | 0.60 – 0.91 | 9.71 | **<0.001** | 0.76 | 0.61 – 0.91 | 9.78 | **<0.001** | | 0.77 | 0.61 – 0.92 | 9.86 | | **<0.001** | | 0.76 | | 0.60 – 0.91 | | 9.66 | | **<0.001** | |
| Negative | 0.68 | 0.49 – 0.86 | 7.21 | **<0.001** | 0.68 | 0.49 – 0.86 | 7.19 | **<0.001** | | 0.68 | 0.50 – 0.87 | 7.16 | | **<0.001** | | 0.68 | | 0.50 – 0.87 | | 7.17 | | **<0.001** | |
| SSEIT OwnEmotion * Positive | -0.22 | -0.37 – -0.07 | -2.90 | **0.004** |  |  |  |  | |  |  |  | |  | |  | |  | |  | |  | |
| SSEIT OwnEmotion * Negative | -0.29 | -0.47 – -0.11 | -3.11 | **0.002** |  |  |  |  | |  |  |  | |  | |  | |  | |  | |  | |
| Positive * Negative | -0.17 | -0.23 – -0.11 | -5.76 | **<0.001** | -0.18 | -0.23 – -0.12 | -5.81 | **<0.001** | | -0.17 | -0.23 – -0.11 | -5.72 | | **<0.001** | | -0.18 | | -0.24 – -0.12 | | -5.78 | | **<0.001** | |
| (SSEIT OwnEmotion * Positive) * Negative | 0.10 | 0.04 – 0.16 | 3.28 | **0.001** |  |  |  |  | |  |  |  | |  | |  | |  | |  | |  | |
| SSEIT OtherEmotion |  |  |  |  | 0.92 | 0.38 – 1.45 | 3.39 | **0.001** | |  |  |  | |  | |  | |  | |  | |  | |
| SSEIT OtherEmotion * Positive |  |  |  |  | -0.18 | -0.32 – -0.03 | -2.35 | **0.019** | |  |  |  | |  | |  | |  | |  | |  | |
| SSEIT OtherEmotion * Negative |  |  |  |  | -0.17 | -0.36 – 0.01 | -1.84 | 0.067 | |  |  |  | |  | |  | |  | |  | |  | |
| (SSEIT OtherEmotion * Positive) * Negative |  |  |  |  | 0.04 | -0.02 – 0.10 | 1.46 | 0.145 | |  |  |  | |  | |  | |  | |  | |  | |
| SSEIT Utilization |  |  |  |  |  |  |  |  | | 0.59 | 0.04 – 1.13 | 2.13 | | **0.034** | |  | |  | |  | |  | |
| SSEIT Utilization * Positive |  |  |  |  |  |  |  |  | | -0.09 | -0.24 – 0.05 | -1.27 | | 0.206 | |  | |  | |  | |  | |
| SSEIT Utilization * Negative |  |  |  |  |  |  |  |  | | -0.08 | -0.27 – 0.11 | -0.84 | | 0.399 | |  | |  | |  | |  | |
| (SSEIT Utilization * Positive) * Negative |  |  |  |  |  |  |  |  | | 0.02 | -0.04 – 0.08 | 0.62 | | 0.538 | |  | |  | |  | |  | |
| SSEIT EmoPerception |  |  |  |  |  |  |  |  | |  |  |  | |  | | 0.78 | | 0.22 – 1.34 | | 2.73 | | **0.006** | |
| SSEIT EmoPerception * Positive |  |  |  |  |  |  |  |  | |  |  |  | |  | | -0.16 | | -0.31 – -0.01 | | -2.04 | | **0.042** | |
| SSEIT EmoPerception * Negative |  |  |  |  |  |  |  |  | |  |  |  | |  | | -0.18 | | -0.37 – 0.01 | | -1.90 | | 0.058 | |
| (SSEIT EmoPerception * Positive) * Negative |  |  |  |  |  |  |  |  | |  |  |  | |  | | 0.05 | | -0.01 – 0.11 | | 1.80 | | 0.073 | |
| **Random Effects** | | | | | | | | | | | | | | | | | | | | | | |  |
| σ^2^ | 0.95 | | | | 0.96 | | | | 0.97 | | | | | | 0.96 | | | | | | | |  |
| τ_00_ | 0.54 _Subject_ | | | | 0.48 _Subject_ | | | | 0.50 _Subject_ | | | | | | 0.51 _Subject_ | | | | | | | |  |
| ICC | 0.36 | | | | 0.33 | | | | 0.34 | | | | | | 0.35 | | | | | | | |  |
| N | 140 _Subject_ | | | | 140 _Subject_ | | | | 140 _Subject_ | | | | | | 140 _Subject_ | | | | | | | |  |
| Observations | 674 | | | | 674 | | | | 674 | | | | | | 674 | | | | | | | |  |
| Marginal R^2^ / Conditional R^2^ | 0.184 / 0.478 | | | | 0.201 / 0.468 | | | | 0.178 / 0.460 | | | | | | 0.187 / 0.469 | | | | | | | |  |
