# Supplementary material for: Individual differences in feelings of certainty surrounding mixed emotions
Source: PLoS One. 2025 Nov 14;20(11):e0332417. doi: 10.1371/journal.pone.0332417 (PMC12617922; doi:10.1371/journal.pone.0332417)
Supplement: S2 Appendix — Significance of predictors determined with t-tests using Satterhwaitte’s method CI = 95% confidence interval. (DOCX) [file pone.0332417.s003.docx]

**Appendix S2: Supplemental Results Tables from Study 2


Certainty of affect predicted by valence and managing one’s own emotions (Study 2)**

|  | **Model 1 (Pos-Neg Interation)** | | | | **Model 2 (Mixed_Griffin)** | | | |
| --- | --- | --- | --- | --- | --- | --- | --- | --- |
| *Predictors* | *Estimates* | *CI* | *Statistic* | *p* | *Estimates* | *CI* | *Statistic* | *p* |
| (Intercept) | 1.84 | 0.00 – 3.68 | 1.97 | **0.050** | 2.59 | 1.17 – 4.01 | 3.58 | **<0.001** |
| Positive | 0.68 | 0.20 – 1.16 | 2.78 | **0.006** | 0.40 | 0.13 – 0.68 | 2.89 | **0.004** |
| Negative | 0.59 | 0.04 – 1.13 | 2.12 | **0.035** | 0.26 | -0.04 – 0.57 | 1.71 | 0.087 |
| SSEIT OwnEmotion | 0.03 | -0.02 – 0.09 | 1.17 | 0.241 | 0.03 | -0.01 – 0.08 | 1.43 | 0.152 |
| Positive * Negative | -0.16 | -0.33 – 0.02 | -1.71 | 0.087 |  |  |  |  |
| Positive * SSEIT OwnEmotion | -0.00 | -0.02 – 0.01 | -0.40 | 0.687 |  |  |  |  |
| Negative * SSEIT OwnEmotion | -0.01 | -0.02 – 0.01 | -0.59 | 0.557 |  |  |  |  |
| (Positive * Negative) * SSEIT OwnEmotion | 0.00 | -0.00 – 0.01 | 0.42 | 0.673 |  |  |  |  |
| Mix |  |  |  |  | -0.16 | -0.38 – 0.05 | -1.48 | 0.138 |
| Mix * SSEIT OwnEmotion |  |  |  |  | -0.00 | -0.01 – 0.01 | -0.23 | 0.822 |
| SSEIT OwnEmotion * Positive |  |  |  |  | -0.00 | -0.01 – 0.01 | -0.49 | 0.621 |
| SSEIT OwnEmotion * Negative |  |  |  |  | -0.00 | -0.01 – 0.01 | -0.39 | 0.696 |
| **Random Effects** | | | | | | | | |
| σ^2^ | 0.73 | | | | 0.72 | | | |
| τ_00_ | 0.39 _Subject_ | | | | 0.37 _Subject_ | | | |
| ICC | 0.35 | | | | 0.34 | | | |
| N | 311 _Subject_ | | | | 311 _Subject_ | | | |
| Observations | 1237 | | | | 1237 | | | |
| Marginal R^2^ / Conditional R^2^ | 0.150 / 0.445 | | | | 0.162 / 0.449 | | | |

**Certainty of affect predicted by valence, meta-mood attention, and repair**

|  | **Model 1 (Attention; Pos-Neg Interaction)** | | | | **Model 2 (Attention; Mixed_Griffin)** | | | | **Model 3 (Repair; Pos-Neg Interaction)** | | | | **Model 4 (Repair; Mixed_Griffin)** | | | |
| --- | --- | --- | --- | --- | --- | --- | --- | --- | --- | --- | --- | --- | --- | --- | --- | --- |
| *Predictors* | *Estimates* | *CI* | *Statistic* | *p* | *Estimates* | *CI* | *Statistic* | *p* | *Estimates* | *CI* | *Statistic* | *p* | *Estimates* | *CI* | *Statistic* | *p* |
| (Intercept) | 2.92 | 2.49 – 3.34 | 13.43 | **<0.001** | 3.65 | 3.35 – 3.95 | 23.78 | **<0.001** | 2.99 | 2.57 – 3.40 | 14.16 | **<0.001** | 3.67 | 3.37 – 3.96 | 24.29 | **<0.001** |
| Positive | 0.60 | 0.49 – 0.70 | 11.21 | **<0.001** | 0.33 | 0.28 – 0.39 | 11.81 | **<0.001** | 0.58 | 0.48 – 0.68 | 11.17 | **<0.001** | 0.33 | 0.28 – 0.39 | 11.94 | **<0.001** |
| Negative | 0.44 | 0.31 – 0.57 | 6.58 | **<0.001** | 0.20 | 0.13 – 0.27 | 5.79 | **<0.001** | 0.41 | 0.28 – 0.54 | 6.28 | **<0.001** | 0.19 | 0.12 – 0.26 | 5.54 | **<0.001** |
| Attention | -0.06 | -0.41 – 0.30 | -0.30 | 0.761 | 0.11 | -0.16 – 0.39 | 0.82 | 0.414 |  |  |  |  |  |  |  |  |
| Positive * Negative | -0.12 | -0.16 – -0.09 | -6.42 | **<0.001** |  |  |  |  | -0.12 | -0.16 – -0.08 | -6.23 | **<0.001** |  |  |  |  |
| Positive * Attention | 0.03 | -0.06 – 0.13 | 0.74 | 0.462 |  |  |  |  |  |  |  |  |  |  |  |  |
| Negative * Attention | 0.08 | -0.03 – 0.19 | 1.37 | 0.170 |  |  |  |  |  |  |  |  |  |  |  |  |
| (Positive * Negative) * Attention | -0.02 | -0.06 – 0.01 | -1.27 | 0.206 |  |  |  |  |  |  |  |  |  |  |  |  |
| Mix |  |  |  |  | -0.19 | -0.24 – -0.15 | -7.98 | **<0.001** |  |  |  |  | -0.19 | -0.24 – -0.14 | -7.81 | **<0.001** |
| Mix * Attention |  |  |  |  | -0.03 | -0.07 – 0.02 | -1.23 | 0.220 |  |  |  |  |  |  |  |  |
| Attention* Positive |  |  |  |  | -0.02 | -0.07 – 0.03 | -0.78 | 0.434 |  |  |  |  |  |  |  |  |
| Attention * Negative |  |  |  |  | 0.03 | -0.03 – 0.09 | 0.86 | 0.390 |  |  |  |  |  |  |  |  |
| Repair |  |  |  |  |  |  |  |  | 0.48 | 0.10 – 0.85 | 2.50 | **0.012** | 0.34 | 0.06 – 0.62 | 2.37 | **0.018** |
| Positive * Repair |  |  |  |  |  |  |  |  | -0.10 | -0.19 – -0.00 | -2.03 | **0.043** |  |  |  |  |
| Negative * Repair |  |  |  |  |  |  |  |  | -0.08 | -0.20 – 0.04 | -1.35 | 0.179 |  |  |  |  |
| (Positive * Negative) * Repair |  |  |  |  |  |  |  |  | 0.03 | -0.01 – 0.07 | 1.60 | 0.110 |  |  |  |  |
| Mix * Repair |  |  |  |  |  |  |  |  |  |  |  |  | 0.04 | -0.01 – 0.09 | 1.64 | 0.101 |
| Repair* Positive |  |  |  |  |  |  |  |  |  |  |  |  | -0.05 | -0.10 – 0.01 | -1.75 | 0.080 |
| Repair* Negative |  |  |  |  |  |  |  |  |  |  |  |  | -0.02 | -0.09 – 0.04 | -0.64 | 0.524 |
| **Random Effects** | | | | | | | | | | | | | | | | |
| σ^2^ | 0.72 | | | | 0.71 | | | | 0.72 | | | | 0.71 | | | |
| τ_00_ | 0.39 _Subject_ | | | | 0.38 _Subject_ | | | | 0.39 _Subject_ | | | | 0.37 _Subject_ | | | |
| ICC | 0.35 | | | | 0.35 | | | | 0.35 | | | | 0.34 | | | |
| N | 305 _Subject_ | | | | 305 _Subject_ | | | | 304 _Subject_ | | | | 304 _Subject_ | | | |
| Observations | 1214 | | | | 1214 | | | | 1209 | | | | 1209 | | | |
| Marginal R^2^ / Conditional R^2^ | 0.135 / 0.439 | | | | 0.148 / 0.444 | | | | 0.161 / 0.453 | | | | 0.176 / 0.459 | | | |

**Certainty of affect predicted by valence and openness**

|  | **Model 1 (Pos-Neg Interaction)** | | | | **Model 2 (Griffin_Mix)** | | | |
| --- | --- | --- | --- | --- | --- | --- | --- | --- |
| *Predictors* | *Estimates* | *CI* | *Statistic* | *p* | *Estimates* | *CI* | *Statistic* | *p* |
| (Intercept) | 2.22 | 0.58 – 3.86 | 2.65 | **0.008** | 2.67 | 1.48 – 3.87 | 4.39 | **<0.001** |
| Positive | 0.62 | 0.19 – 1.04 | 2.86 | **0.004** | 0.41 | 0.19 – 0.64 | 3.55 | **<0.001** |
| Negative | 0.56 | 0.06 – 1.06 | 2.21 | **0.027** | 0.39 | 0.13 – 0.65 | 2.91 | **0.004** |
| Openness | 0.13 | -0.17 – 0.44 | 0.85 | 0.398 | 0.18 | -0.04 – 0.40 | 1.57 | 0.116 |
| Positive * Negative | -0.12 | -0.27 – 0.03 | -1.54 | 0.123 |  |  |  |  |
| Positive * Openness | -0.00 | -0.08 – 0.07 | -0.12 | 0.908 |  |  |  |  |
| Negative * Openness | -0.03 | -0.12 – 0.07 | -0.55 | 0.580 |  |  |  |  |
| (Positive * Negative) * Openness | 0.00 | -0.03 – 0.03 | 0.02 | 0.981 |  |  |  |  |
| Mix |  |  |  |  | -0.24 | -0.43 – -0.04 | -2.38 | **0.018** |
| Mix * Openness |  |  |  |  | 0.01 | -0.03 – 0.05 | 0.52 | 0.601 |
| Openness * Positive |  |  |  |  | -0.01 | -0.06 – 0.03 | -0.62 | 0.537 |
| Openness * Negative |  |  |  |  | -0.04 | -0.09 – 0.01 | -1.44 | 0.150 |
| **Random Effects** | | | | | | | | |
| σ^2^ | 0.73 | | | | 0.72 | | | |
| τ_00_ | 0.40 _Subject_ | | | | 0.39 _Subject_ | | | |
| ICC | 0.35 | | | | 0.35 | | | |
| N | 311 _Subject_ | | | | 311 _Subject_ | | | |
| Observations | 1237 | | | | 1237 | | | |
| Marginal R^2^ / Conditional R^2^ | 0.134 / 0.440 | | | | 0.148 / 0.446 | | | |

**Certainty of affect predicted by valence and extraversion**

|  | **Model 1 (Pos-Neg Interaction)** | | | | **Model 2 (Griffin_Mix)** | | | |
| --- | --- | --- | --- | --- | --- | --- | --- | --- |
| *Predictors* | *Estimates* | *CI* | *Statistic* | *p* | *Estimates* | *CI* | *Statistic* | *p* |
| (Intercept) | 3.02 | 2.22 – 3.81 | 7.40 | **<0.001** | 3.48 | 2.89 – 4.07 | 11.53 | **<0.001** |
| Positive | 0.57 | 0.37 – 0.77 | 5.54 | **<0.001** | 0.39 | 0.28 – 0.50 | 6.88 | **<0.001** |
| Negative | 0.34 | 0.10 – 0.58 | 2.74 | **0.006** | 0.18 | 0.05 – 0.31 | 2.69 | **0.007** |
| Extraversion | -0.05 | -0.30 – 0.20 | -0.39 | 0.696 | 0.03 | -0.15 – 0.21 | 0.34 | 0.731 |
| Positive * Negative | -0.09 | -0.16 – -0.02 | -2.40 | **0.016** |  |  |  |  |
| Positive * Extraversion | 0.01 | -0.05 – 0.07 | 0.40 | 0.690 |  |  |  |  |
| Negative * Extraversion | 0.03 | -0.04 – 0.10 | 0.89 | 0.373 |  |  |  |  |
| (Positive * Negative) * Extraversion | -0.01 | -0.03 – 0.01 | -0.96 | 0.337 |  |  |  |  |
| Mix |  |  |  |  | -0.14 | -0.24 – -0.05 | -3.05 | **0.002** |
| Mix * Extraversion |  |  |  |  | -0.01 | -0.04 – 0.01 | -1.09 | 0.277 |
| Extraversion * Positive |  |  |  |  | -0.01 | -0.05 – 0.02 | -0.79 | 0.429 |
| Extraversion * Negative |  |  |  |  | 0.01 | -0.03 – 0.05 | 0.48 | 0.631 |
| **Random Effects** | | | | | | | | |
| σ^2^ | 0.73 | | | | 0.72 | | | |
| τ_00_ | 0.40 _Subject_ | | | | 0.39 _Subject_ | | | |
| ICC | 0.35 | | | | 0.35 | | | |
| N | 310 _Subject_ | | | | 310 _Subject_ | | | |
| Observations | 1233 | | | | 1233 | | | |
| Marginal R^2^ / Conditional R^2^ | 0.129 / 0.437 | | | | 0.142 / 0.441 | | | |

**Certainty of affect predicted by valence and emotional stability**

|  | **Model 1 (Pos-Neg Interaction)** | | | | **Model 2 (Griffin_Mix)** | | | |
| --- | --- | --- | --- | --- | --- | --- | --- | --- |
| *Predictors* | *Estimates* | *CI* | *Statistic* | *p* | *Estimates* | *CI* | *Statistic* | *p* |
| (Intercept) | 1.94 | 0.79 – 3.09 | 3.32 | **0.001** | 3.05 | 2.20 – 3.90 | 7.05 | **<0.001** |
| Positive | 0.70 | 0.42 – 0.99 | 4.82 | **<0.001** | 0.33 | 0.18 – 0.49 | 4.18 | **<0.001** |
| Negative | 0.72 | 0.37 – 1.06 | 4.09 | **<0.001** | 0.34 | 0.15 – 0.52 | 3.47 | **0.001** |
| EmoStable | 0.21 | -0.03 – 0.46 | 1.71 | 0.088 | 0.11 | -0.07 – 0.30 | 1.23 | 0.218 |
| Positive * Negative | -0.17 | -0.27 – -0.07 | -3.39 | **0.001** |  |  |  |  |
| Positive * EmoStable | -0.03 | -0.09 – 0.03 | -0.83 | 0.409 |  |  |  |  |
| Negative * EmoStable | -0.07 | -0.14 – 0.01 | -1.74 | 0.083 |  |  |  |  |
| (Positive * Negative) * EmoStable | 0.01 | -0.01 – 0.04 | 1.20 | 0.231 |  |  |  |  |
| Mix |  |  |  |  | -0.24 | -0.37 – -0.10 | -3.51 | **<0.001** |
| Mix * EmoStable |  |  |  |  | 0.01 | -0.02 – 0.04 | 0.84 | 0.402 |
| EmoStable * Positive |  |  |  |  | 0.00 | -0.03 – 0.04 | 0.21 | 0.836 |
| EmoStable * Negative |  |  |  |  | -0.03 | -0.07 – 0.01 | -1.36 | 0.175 |
| **Random Effects** | | | | | | | | |
| σ^2^ | 0.73 | | | | 0.72 | | | |
| τ_00_ | 0.39 _Subject_ | | | | 0.38 _Subject_ | | | |
| ICC | 0.35 | | | | 0.34 | | | |
| N | 310 _Subject_ | | | | 310 _Subject_ | | | |
| Observations | 1233 | | | | 1233 | | | |
| Marginal R^2^ / Conditional R^2^ | 0.144 / 0.443 | | | | 0.156 / 0.447 | | | |

**Certainty of affect predicted by valence and agreeableness**

|  | **Model 1 (Pos-Neg Interaction)** | | | | **Model 2 (Griffin_Mix)** | | | |
| --- | --- | --- | --- | --- | --- | --- | --- | --- |
| *Predictors* | *Estimates* | *CI* | *Statistic* | *p* | *Estimates* | *CI* | *Statistic* | *p* |
| (Intercept) | 1.16 | -0.44 – 2.76 | 1.43 | 0.154 | 2.18 | 1.01 – 3.35 | 3.66 | **<0.001** |
| Positive | 0.86 | 0.45 – 1.28 | 4.08 | **<0.001** | 0.50 | 0.28 – 0.73 | 4.45 | **<0.001** |
| Negative | 0.78 | 0.27 – 1.29 | 3.01 | **0.003** | 0.42 | 0.14 – 0.69 | 2.97 | **0.003** |
| Agreeableness | 0.35 | 0.04 – 0.66 | 2.21 | **0.027** | 0.28 | 0.06 – 0.51 | 2.47 | **0.014** |
| Positive * Negative | -0.18 | -0.33 – -0.02 | -2.24 | **0.025** |  |  |  |  |
| Positive * Agreeableness | -0.05 | -0.13 – 0.02 | -1.35 | 0.176 |  |  |  |  |
| Negative * Agreeableness | -0.07 | -0.17 – 0.03 | -1.40 | 0.163 |  |  |  |  |
| (Positive * Negative) * Agreeableness | 0.01 | -0.02 – 0.04 | 0.78 | 0.438 |  |  |  |  |
| Mix |  |  |  |  | -0.25 | -0.45 – -0.05 | -2.42 | **0.016** |
| Mix * Agreeableness |  |  |  |  | 0.01 | -0.03 – 0.05 | 0.60 | 0.549 |
| Agreeableness * Positive |  |  |  |  | -0.03 | -0.07 – 0.01 | -1.51 | 0.132 |
| Agreeableness * Negative |  |  |  |  | -0.04 | -0.10 – 0.01 | -1.54 | 0.124 |
| **Random Effects** | | | | | | | | |
| σ^2^ | 0.73 | | | | 0.72 | | | |
| τ_00_ | 0.39 _Subject_ | | | | 0.38 _Subject_ | | | |
| ICC | 0.35 | | | | 0.34 | | | |
| N | 311 _Subject_ | | | | 311 _Subject_ | | | |
| Observations | 1237 | | | | 1237 | | | |
| Marginal R^2^ / Conditional R^2^ | 0.147 / 0.444 | | | | 0.159 / 0.448 | | | |

**Certainty of affect predicted by valence and conscientiousness**

|  | **Model 1 (Pos-Neg Interaction)** | | | | **Model 2 (Griffin_Mix)** | | | |
| --- | --- | --- | --- | --- | --- | --- | --- | --- |
| *Predictors* | *Estimates* | *CI* | *Statistic* | *p* | *Estimates* | *CI* | *Statistic* | *p* |
| (Intercept) | 0.92 | -0.63 – 2.48 | 1.17 | 0.243 | 2.32 | 1.19 – 3.44 | 4.03 | **<0.001** |
| Positive | 0.93 | 0.54 – 1.32 | 4.71 | **<0.001** | 0.46 | 0.25 – 0.67 | 4.31 | **<0.001** |
| Negative | 0.96 | 0.50 – 1.43 | 4.08 | **<0.001** | 0.49 | 0.25 – 0.74 | 3.93 | **<0.001** |
| Conscientious | 0.38 | 0.09 – 0.66 | 2.60 | **0.010** | 0.24 | 0.04 – 0.45 | 2.30 | **0.022** |
| Positive * Negative | -0.22 | -0.36 – -0.08 | -3.11 | **0.002** |  |  |  |  |
| Positive * Conscientious | -0.06 | -0.13 – 0.01 | -1.76 | 0.079 |  |  |  |  |
| Negative * Conscientious | -0.10 | -0.19 – -0.02 | -2.37 | **0.018** |  |  |  |  |
| (Positive * Negative) * Conscientious | 0.02 | -0.01 – 0.04 | 1.45 | 0.147 |  |  |  |  |
| Mix |  |  |  |  | -0.30 | -0.47 – -0.12 | -3.35 | **0.001** |
| Mix * Conscientious |  |  |  |  | 0.02 | -0.01 – 0.05 | 1.28 | 0.200 |
| Conscientious * Positive |  |  |  |  | -0.02 | -0.06 – 0.02 | -1.12 | 0.262 |
| Conscientious * Negative |  |  |  |  | -0.06 | -0.10 – -0.01 | -2.41 | **0.016** |
