# Supplementary material for: Individual differences in feelings of certainty surrounding mixed emotions
Source: PLoS One. 2025 Nov 14;20(11):e0332417. doi: 10.1371/journal.pone.0332417 (PMC12617922; doi:10.1371/journal.pone.0332417)
Supplement: S3 Appendix — Each subject rated 4–5 videos. (DOCX) [file pone.0332417.s004.docx]

**Appendix S3: Stimuli Ratings Across Both Studies**

**Mean (and standard deviation) valence, arousal, emotional certainty, and familiarity ratings for video stimuli**

| **Video Title** | **Positive Valence (1-5)** | **Negative Valence (1-5)** | **Arousal (1-5)** | **Emotional Certainty (1-6)** | **Familiarity (1-4)** |
| --- | --- | --- | --- | --- | --- |
| 1 Small Step | 3.93±1.02 | 2.07±0.88 | 2.88±1.13 | 5.41±0.96 | 1.07±0.32 |
| 500 Days of Summer | 2.73±0.99 | 2.46±1.04 | 2.16±0.99 | 4.38±1.27 | 1.39±0.76 |
| Bao | 3.51±1.27 | 2.1±1.05 | 2.83±1.04 | 4.85±1.16 | 1.81±1.05 |
| Big Fish | 3.4±1.21 | 2.43±1.23 | 2.57±1.19 | 4.66±1.33 | 1.41±0.88 |
| Butera | 3.04±1.1 | 3.16±1.15 | 2.95±1.16 | 4.76±1.22 | 1.02±0.13 |
| Eternal Sunshine of the Spotless Mind | 2.25±0.82 | 2.92±1.09 | 2.54±1.16 | 4.22±1.46 | 1.31±0.73 |
| Fluke | 3.11±1.29 | 2.44±1.3 | 2.54±0.97 | 4.79±1.2 | 1.13±0.43 |
| Hair Love | 4.33±0.76 | 1.36±0.63 | 2.50±0.97 | 5.34±0.8 | 1.17±0.46 |
| The King and the Pawn | 2.95±1.07 | 2.95±1.05 | 2.73±1.10 | 4.8±1.34 | 1±0 |
| Kitbull | 3.76±1.27 | 2.67±1.26 | 3.14±1.23 | 5.24±1.19 | 1.06±0.24 |
| Late Afternoon | 3.44±1.1 | 2.26±1.11 | 2.71±1.16 | 4.87±1.22 | 0.98±0.13 |
| Life of Death | 2.02±1 | 3.25±1.24 | 2.13±1.03 | 5.12±1.15 | 1.05±0.22 |
| Lost and Found | 3.23±1.05 | 2.5±1.2 | 3.05±1.14 | 4.93±1.27 | 1.12±0.49 |
| Me Before You | 3.03±1.17 | 1.9±0.98 | 1.95±0.97 | 4.81±1.25 | 1.2±0.58 |
| Miles to Fly | 4.12±0.77 | 1.83±0.97 | 2.70±1.09 | 5.17±1.08 | 1.08±0.33 |
| Monster's Inc. | 3.96±1.07 | 1.93±1.07 | 2.77±1.28 | 5.46±0.83 | 2.6±1.16 |
| Neila | 3.02±0.9 | 2.46±0.98 | 2.89±0.94 | 4.73±1.24 | 1.11±0.49 |
| Out of Sight | 3.89±1.02 | 1.59±0.84 | 2.98±1.11 | 5.15±1.04 | 1.11±0.47 |
| Pip | 4.38±0.83 | 1.37±0.68 | 3.17±1.14 | 5.52±0.72 | 1.27±0.7 |
| Present | 3.88±1 | 2.25±1.06 | 2.73±0.94 | 5.22±1.07 | 1.14±0.39 |
| Purl | 3.63±1.12 | 2.42±1.15 | 2.75±1.12 | 4.9±1.2 | 1.19±0.51 |
| The Queen's Gambit | 2.65±1.05 | 2.63±1.11 | 2.42±1.04 | 4.45±1.41 | 1.42±0.63 |
| Roman Holiday | 2.63±0.96 | 2.38±0.92 | 2.12±1.04 | 4.3±1.42 | 1.15±0.52 |
| The Box | 4.1±0.78 | 1.43±0.78 | 2.49±1.06 | 5.02±1.13 | 1.05±0.21 |
| Toy Story 3 | 3.67±1.05 | 1.8±1.01 | 2.73±1.09 | 5.28±0.78 | 2.17±0.99 |
| Up | 3.27±1.19 | 2.47±1.29 | 2.90±1.12 | 5.18±1.08 | 2.28±1.06 |
| Wedding Cake | 2.63±1.15 | 2.88±1.32 | 2.73±1.13 | 4.83±1.1 | 1.05±0.29 |
| Zero | 3.02±1.24 | 2.82±1.06 | 2.75±1.17 | 4.61±1.41 | 1.2±0.59 |

**Categorical emotion labels chosen by subjects in response to video stimuli**

| **Video Title** | **Happy** | **Excited** | **Amused** | **Love** | **Fearful** | **Sad** | **Angry** | **Disgust** | **Nostalgic** | **Bittersweet** | **Awe** | **Conflicted** |
| --- | --- | --- | --- | --- | --- | --- | --- | --- | --- | --- | --- | --- |
| 1 Small Step | 76.92% | 30.77% | 30.77% | 46.15% | 38.46% | 76.92% | 7.69% | 0.00% | 76.92% | 61.54% | 46.15% | 38.46% |
| 500 Days of Summer | 50.00% | 14.29% | 35.71% | 35.71% | 21.43% | 57.14% | 7.14% | 14.29% | 35.71% | 64.29% | 7.14% | 50.00% |
| Bao | 61.54% | 46.15% | 23.08% | 30.77% | 0.00% | 23.08% | 0.00% | 7.69% | 38.46% | 61.54% | 15.38% | 23.08% |
| Big Fish | 64.29% | 42.86% | 21.43% | 64.29% | 14.29% | 71.43% | 0.00% | 0.00% | 14.29% | 71.43% | 7.14% | 28.57% |
| Butera | 75.00% | 41.67% | 0.00% | 0.00% | 75.00% | 58.33% | 41.67% | 41.67% | 16.67% | 25.00% | 25.00% | 66.67% |
| Eternal Sunshine of the Spotless Mind | 42.86% | 14.29% | 21.43% | 35.71% | 35.71% | 42.86% | 7.14% | 0.00% | 7.14% | 35.71% | 14.29% | 14.29% |
| Fluke | 87.50% | 12.50% | 31.25% | 81.25% | 0.00% | 56.25% | 0.00% | 0.00% | 6.25% | 68.75% | 50.00% | 6.25% |
| Hair Love | 73.33% | 26.67% | 0.00% | 46.67% | 20.00% | 66.67% | 20.00% | 6.67% | 26.67% | 46.67% | 20.00% | 40.00% |
| The King and the Pawn | 100.00% | 71.43% | 14.29% | 78.57% | 64.29% | 92.86% | 57.14% | 57.14% | 21.43% | 42.86% | 42.86% | 28.57% |
| Kitbull | 68.75% | 18.75% | 18.75% | 62.50% | 18.75% | 75.00% | 6.25% | 0.00% | 81.25% | 81.25% | 25.00% | 31.25% |
| Late Afternoon | 69.23% | 38.46% | 30.77% | 23.08% | 46.15% | 30.77% | 30.77% | 7.69% | 7.69% | 46.15% | 30.77% | 46.15% |
| Life of Death | 25.00% | 0.00% | 8.33% | 50.00% | 41.67% | 75.00% | 8.33% | 0.00% | 16.67% | 50.00% | 16.67% | 25.00% |
| Lost and Found | 68.75% | 50.00% | 56.25% | 68.75% | 31.25% | 75.00% | 6.25% | 6.25% | 6.25% | 62.50% | 31.25% | 18.75% |
| Me Before You | 33.33% | 0.00% | 0.00% | 73.33% | 0.00% | 60.00% | 0.00% | 0.00% | 66.67% | 66.67% | 20.00% | 33.33% |
| Miles to Fly | 73.33% | 40.00% | 13.33% | 53.33% | 13.33% | 60.00% | 0.00% | 13.33% | 33.33% | 66.67% | 20.00% | 33.33% |
| Monster's Inc. | 78.57% | 42.86% | 42.86% | 64.29% | 0.00% | 71.43% | 7.14% | 0.00% | 78.57% | 64.29% | 28.57% | 7.14% |
| Neila | 92.86% | 42.86% | 42.86% | 71.43% | 28.57% | 64.29% | 14.29% | 0.00% | 21.43% | 50.00% | 50.00% | 28.57% |
| Out of Sight | 78.57% | 57.14% | 42.86% | 28.57% | 14.29% | 35.71% | 7.14% | 7.14% | 14.29% | 28.57% | 35.71% | 28.57% |
| Pip | 100.00% | 43.75% | 56.25% | 50.00% | 12.50% | 50.00% | 0.00% | 0.00% | 12.50% | 37.50% | 68.75% | 18.75% |
| Present | 71.43% | 21.43% | 21.43% | 57.14% | 14.29% | 57.14% | 71.43% | 7.14% | 7.14% | 35.71% | 21.43% | 42.86% |
| Purl | 81.25% | 50.00% | 43.75% | 12.50% | 12.50% | 43.75% | 62.50% | 31.25% | 0.00% | 37.50% | 18.75% | 43.75% |
| The Queen's Gambit | 35.29% | 35.29% | 5.88% | 17.65% | 17.65% | 58.82% | 11.76% | 0.00% | 35.29% | 41.18% | 5.88% | 41.18% |
| Roman Holiday | 37.50% | 25.00% | 25.00% | 62.50% | 12.50% | 50.00% | 0.00% | 0.00% | 37.50% | 37.50% | 6.25% | 50.00% |
| The Box | 88.24% | 17.65% | 82.35% | 47.06% | 5.88% | 23.53% | 0.00% | 5.88% | 29.41% | 35.29% | 29.41% | 29.41% |
| Toy Story 3 | 66.67% | 16.67% | 25.00% | 58.33% | 0.00% | 91.67% | 8.33% | 0.00% | 100.00% | 91.67% | 16.67% | 66.67% |
| Up | 62.50% | 37.50% | 31.25% | 75.00% | 25.00% | 81.25% | 6.25% | 0.00% | 68.75% | 75.00% | 12.50% | 18.75% |
| Wedding Cake | 60.00% | 40.00% | 20.00% | 53.33% | 20.00% | 53.33% | 60.00% | 33.33% | 0.00% | 40.00% | 0.00% | 80.00% |
| Zero | 71.43% | 42.86% | 28.57% | 28.57% | 50.00% | 64.29% | 35.71% | 28.57% | 7.14% | 7.14% | 28.57% | 28.57% |
